# Allergic Rhinitis-Induced Anxiety and Depression: An Autobiographical Case Report

**DOI:** 10.7759/cureus.36560

**Published:** 2023-03-23

**Authors:** Saranya Ramsridhar

**Affiliations:** 1 Department of Oral Pathology, Sathyabama Dental College and Hospital, Sathyabama Institute of Science and Technology, Chennai, IND

**Keywords:** rhinorrhea, comorbidities, yoga, quality of life, anxiety, depression, congestion, sneezing, pruritis, global health concern

## Abstract

Allergic rhinitis (AR) formerly perceived to be a nuisance disease is of global health concern now, causing significant economic and social burden worldwide. It is a common inflammatory condition of the nasal mucosa characterized by four cardinal symptoms: nasal pruritus, sneezing, rhinorrhea, and nasal congestion. Poorly controlled AR can also lead to impairment in sleep and a decrease in school/work performance, thereby affecting the quality of life. In addition, AR can produce serious mental and psychological disorders such as depression and anxiety. Yoga can be used as an alternative therapy to treat AR as it has been proven to have beneficial effects in alleviating the symptoms of AR and can also have an overall relaxing effect on the body and mind. The aim of this case report is to share my first-hand experience of enduring endless suffering due to AR because of my negligent behavior. My chronic symptoms that did not resolve on medication led me to anxiety and depression, and I finally resorted to yoga and meditation to battle the disorder.

## Introduction

Allergic rhinitis (AR), colloquially known as hay fever, is an atopic disorder affecting around 400 million people globally and is caused by an immunoglobulin E (IgE)-mediated reaction to inhaled allergens in sensitized individuals. The majority of its symptoms are seen before the age of 20, with a peak incidence between 20 and 40 years, and symptoms gradually subside after the age of 40. It shows a male predilection in childhood and a female predilection in adolescents [1]. Historically, AR was considered to be localized to the upper respiratory tract, but current research indicates that AR is more of a systemic airway disease involving the upper and lower respiratory tract as it frequently coexists with asthma [2].

In AR, the allergic response is seen as early- and late-phase reactions triggered by exposure to allergens such as pollen, dust mites, and/or animal dander. In the early phase, mast cells undergo degranulation, releasing a variety of mediators, such as histamine, which rapidly induces nasal symptoms such as sneezing and rhinorrhea and ocular symptoms such as itching, redness, and watering. Late-phase reaction develops hours after the initial response, with the arrival of cytokines, prostaglandins, and leukotrienes that promote the infiltration of eosinophils, neutrophils, basophils, lymphocytes, and monocytes into the nasal mucosa, producing nasal edema with resultant congestion. Bronchial hyperresponsiveness occurs as a result of mucosal inflammation, and the tissue now becomes primed and reacts more vigorously to even normal stimuli [3].

Earlier, AR has been categorized as seasonal or perennial, but now, according to Allergic Rhinitis and Its Impact on Asthma (ARIA) guidelines, AR is classified as “intermittent” if symptoms are present less than four days per week or for less than four consecutive weeks and “persistent” if symptoms are present more than four days per week and for more than four consecutive weeks. Symptoms are classified as mild when there is no sleep impairment and the patient is able to perform normal activities and moderate/severe when sleep or daily activities are affected [2]. AR, when left untreated, is often associated with significant short- and long-term complications, which include nasal polyps, acute and chronic sinusitis, asthma, eustachian tube dysfunction/otitis media, sleep apnea, decreased cognitive functioning, decreased long-term productivity, and craniofacial abnormalities [4].

AR can also have a serious impact on mental health, leading to depression and anxiety that not only complicates the treatment but also affects the quality of life of patients, thus adding to the burden on families and society [5]. The severity and duration of symptoms must be considered while treating AR, and treatment options include patient education; pharmacotherapy options, such as nasal/oral glucocorticoids, antihistamines, leukotriene receptor antagonists, mast cell membrane stabilizers, decongestants, and anticholinergic drugs; allergen-specific immunotherapy; and biologics such as omalizumab and dupilumab [6]. In this case report, I will share my experience of AR with the main purpose of creating awareness about early diagnosis and the psychological impact of the disease to prevent associated comorbidities and improve quality of life.

## Case presentation

I am a 34-year-old dentist working as an associate professor at a university for the past five years. I have been experiencing severe sneezing for the past six years along with a runny nose and nasal congestion. I have no family history of allergies, and I do not suffer from any other medical illness, but I have undergone a lot of emotional stress on the personal front. My symptoms have been more severe on waking up early in the morning. Although I have no trouble falling asleep, I often wake up in the middle of the night due to an itchy nose that triggers my sneezing, following which I sneeze continuously that can last for several minutes, and it is usually accompanied by clear thick nasal discharge. I also face unilateral nasal congestion, especially while lying down flat on my back during bedtime. Cold temperatures such as sleeping in a closed air-conditioned room every day owing to the hot humid temperature outside exacerbated my symptoms. My sneezing was aggravated by the hair my pet dogs shed or whenever the house is dusty and unclean.

Due to disturbed sleep at night, I experienced daytime drowsiness, leaving me feeling irritable and tired. Being a doctor, I was about to self-diagnose that I was suffering from AR, but I did not seek any professional help and continued to ignore my symptoms. Whenever my condition worsened, I opted for self-medication that provided me temporary relief but left me feeling groggy throughout the day. Steam inhalation and warm showers helped me with my nasal congestion but only to a certain extent.

In due course, my symptoms started worsening, and I started sneezing all year round, almost every other day, and at times, it was so severe that I sneezed nearly 100 times a day. My condition became more noticeable among others, leaving me feeling embarrassed most of the time. I mostly experienced a feeling of heaviness as soon as I woke up and also had frequent headaches. My nasal congestion became so severe that I had difficulty breathing through my nose and started to mouth breath. Dark circles started forming around my eyes, and the skin around my nostrils became dry. I also experienced occasional epistaxis and ear blockage.

There were several days when my sneezing and nasal congestion were at its extreme that I barely slept for a few hours. Since the quality of my sleep was affected, my energy and concentration levels started decreasing, my work efficiency was reduced, and I also failed to report to work most of the time. My persistent AR took a major toll on my mental health, and I started feeling stressed and depressed, started reducing all my social interactions, and isolated myself. As a doctor, I know that increased stress levels can trigger allergies and that allergies can further lead to more stress, thus forming a vicious cycle.

At one point, I was forced by my family and friends to seek medical help, and I consulted an ENT specialist. After a thorough physical examination, he confirmed my self-diagnosis of AR and stated that my anterior nasal mucosa was ulcerated and prescribed a nasal cream for the same (morning and night inside both nostrils), which is a combination of chlorhexidine hydrochloride and neomycin sulfate. I was also prescribed a combination of montelukast and fexofenadine (one tablet at night after food) and a nasal spray (two puffs to each nostril thrice daily) that contains sodium chloride as the active ingredient to alleviate my clinical symptoms of AR. I was asked to follow all this for a week and report back for review.

During the time of treatment and for a brief period afterward, I felt better, but to my dismay, my happiness was short-lived as my allergy started cropping up again. I switched to Ayurvedic treatment, where I was recommended herbal oil massages and various kashyamas to balance my doshas, only to leave me feeling disappointed once again as my symptoms did not resolve. Frustrated as I was unable to find any cure and my anxiety is at its peak now, I finally resorted to yoga, which includes asanas, pranayama, or breathing exercises along with meditation. I routinely did vrikshasana (tree pose), veerabhadrasana (warrior pose), trikosana (triangle pose), salamba sarvangasana (shoulder-stand pose), and matsyasana (fish pose) along with surya namaskar under the guidance of a trained yoga practitioner. These asanas were highly recommended by my trainer to relieve AR. I also practiced pranayama, which basically involves voluntary inhalation, retention, and exhalation of breath that will help in clearing nasal passages and will also improve lung capacity. In addition to the above, I meditated for around 15 minutes every day, which helped immensely in calming my mind by easing my stress. I kept my bedroom clean and tidy and pet-free. I started sleeping with my head propped up on a high pillow as sinuses can drain more properly this way.

Yoga not only helped keep my stress at bay but also slowly improved my condition, quality of sleep, focus, and concentration levels, and I was able to perform better at work. Although I was one of the unlucky many to suffer from AR, I learned to deal with it by choosing yoga as an alternative therapy, and this decision was purely autonomous, but what worked for me might not work for everyone else. Nosing around the different treatment options available helped my nose function smoothly, yet I have not completely got rid of this illness and occasionally experience symptoms, but over time, AR had a progressively less negative effect on my overall physical and mental status, and I am a stronger, happier person now.

## Discussion

Although being highly prevalent, AR often goes undetected due to its long-standing nature and patients failing to recognize the impact of this illness on their daily lives. Hence, patients with unrecognized AR not seeking medical treatment or those receiving inappropriate treatment pose the greatest global burden of this illness. This case history describes many of the AR-related challenges mentioned in the literature that are discussed below.

Individuals with persistent emotional stress have frequent allergy flares, and stress and anxiety can enhance and prolong allergy symptoms. Psychological stress is strongly associated with persistent AR, and when stress is kept under control by pharmacotherapy of imipramine and levocetirizine, AR symptoms improved, resulting in a better quality of life [7-9]. Psychological stress also intensifies mast cell-dependent inflammation, inducing nasal allergy. The primary stress-mediating neurohormone, the corticotropin-releasing stress hormone (CRH), plays a key role in stress-induced nasal allergy. This hypothesis was probed by a team of researchers who stimulated CRH by adding it to a nasal polyp organ culture, which substantially increased the number of mast cells in the human nasal mucosa, which plays a central role in the initiation of an allergic immune response [10]. Thus, the influence goes both ways: not only can allergies cause stress, but stress can make allergies worse. In my case, dealing with a lot of emotional stress due to personal issues could also be a contributing factor in developing AR.

Jaruvongvanich et al. [11], in their study, reported that the 10 most common symptoms of moderately severe AR are nasal congestion, rhinorrhea, sneeze, itchy nose, fatigue, mouth breathing, daytime somnolence, postnasal drip, itchy eyes, and dry mouth. Stasis of blood secondary to pressure on the veins from edema of the mucous membranes of the nasal and paranasal cavities produces dark, swollen circles under the eyes known as allergic shiners [12]. Irritation of the nasal mucous membrane from chronic AR or “allergic salute” (nasal rubbing), a known cause of trauma to the nose, results in frequent epistaxis [13]. The already increased nasal airway resistance in AR patients, which occurs because of nasal congestion, almost triples when lying in a horizontal position than upright, and hence, symptoms are severe on lying down in AR individuals. Also, in AR, symptoms such as sneezing, nasal congestion, and rhinorrhea can be extreme on waking as the levels of inflammatory cells are the highest in the early morning [14]. All these clinical signs and symptoms correlated with the present case report.

Shedden [15] reported nasal congestion to be the most bothersome symptom in AR in an online survey conducted on AR individuals. The majority of the participants with nasal congestion reported sleep disturbance, lack of concentration, poor productivity at school/work, and inability to perform daily activities. According to this survey, nasal congestion also left many of the participants feeling uncomfortable, frustrated, and having to slow down in the morning. Robles-Figueroa et al. [16], in their cross-sectional study, reported a loss of energy and concentration difficulty more frequently in AR patients compared to healthy individuals.

Symptoms such as nasal itching/congestion and olfactory disturbances are attributed to the development of depression in AR. AR patients with comorbidities such as asthma and sinusitis are more prone to depression and suicidal behavior. Excessive fear regarding the side effects of the drugs and sometimes the medication itself can cause depression in AR. There is some correlation between maternal inheritance and the *ADCYAP1R1* gene in association with depression in AR. In AR, when an IgE-mediated immune response is triggered by allergens, peripheral inﬂammatory signals or cytokines such as interleukin-1 beta (IL-1β), IL-6, tumor necrosis factor-alpha (TNF-α), granulocyte-macrophage colony-stimulating factor (GM-CSF), IL-5, IL-13, and IL-4 in the nose enters the central system through neural (olfactory and trigeminal nerves), cellular, and humoral pathways, causing neuroinﬂammation, oxidative stress, and neurotransmitter disturbances in the brain, which results in depression [5].

Medications for AR can only provide symptomatic relief, but the improvement is not long-lasting as in my case. Immunotherapy might show better efficacy but is very expensive, which adds to the economic burden of developing countries. Holistic approaches such as yoga are mind-body therapies that are cost-effective and have no side effects. Recent evidence has proven that yoga can be a potential alternative or complementary treatment for AR.

Yoga reduces nasal congestion and improves lung capacity, nasal airflow, and symptom score in AR. It also reduces psychological stress and sleep difficulty. It may downregulate certain inflammatory and pro-inflammatory cytokines and upregulate anti-inflammatory interleukin in AR. Yoga might also reduce the hyperresponsiveness of the nasal airway by improving sympathovagal balance. Hatha yoga is a combination of postural exercise (asana), deep breathing (pranayama), and meditation (shavasana) [17]. Chanta et al. [18], in their study, reported that eight weeks of hatha yoga training increased the nasal inspiratory flow and decreased rhinitis symptoms and nasal blood flow in the yoga group. Also, the yoga group had significantly higher nasal secretion of IL-2 compared to the control group. Chellaa et al. [19], in their study, proved a significant decrease in the total nasal airway resistance in both healthy volunteers and AR patients, and the pulmonary function parameters also showed improvement in both groups after regular yoga practice. The quality of life of the individuals was also evaluated to be better [17-19]. Yoga can hence be used as an adjuvant therapy to standard medical therapy for better outcomes in AR, but further studies in the future would be beneficial to evaluate this.

## Conclusions

AR can either be a seasonal nuisance or a year-round hassle, but it should never be waived off as trivial with its symptoms being overlooked or undertreated. Patients often underestimate the severity of this debilitating condition and fail to seek medical therapy, which can turn out to be detrimental to physical and mental health. Unfortunately, there is no permanent cure for AR, but the effects of the disease can be lessened with accurate and timely diagnosis. Physicians should acknowledge the complexity of the condition in AR patients, and a multidisciplinary approach along with psychological support not only provides symptom relief and controls associated comorbidities but can also improve the quality of life of AR patients.
